# Larval crowding enhances dengue virus loads in *Aedes aegypti*, a relationship that might increase transmission in urban environments

**DOI:** 10.1371/journal.pntd.0012482

**Published:** 2024-09-10

**Authors:** Heverton L. C. Dutra, Dustin J. Marshall, Belinda Comerford, Brianna P. McNulty, Anastacia M. Diaz, Matthew J. Jones, Austin J. Mejia, Ottar N. Bjornstad, Elizabeth A. McGraw

**Affiliations:** 1 The Biology Department, The Pennsylvania State University, University Park, Pennsylvania, United States of America; 2 The Center for Infectious Disease Dynamics, The Huck Institutes of the Life Sciences, The Pennsylvania State University, University Park, Pennsylvania, United States of America; 3 The School of Life Sciences, Monash University, Melbourne, Australia; 4 The Entomology Department, The Pennsylvania State University, University Park, Pennsylvania, United States of America; University of Wisconsin Madison, UNITED STATES OF AMERICA

## Abstract

**Background:**

Climate change and urbanization will alter the global distribution of disease vectors, changing the disease burden in yet unpredictable ways. *Aedes aegypti* is a mosquito responsible for transmitting dengue, Zika, chikungunya, and yellow fever viruses that breeds in containers associated with urban environments. We sought to understand how ambient temperature and larval densities in the immature aquatic phases determine adult life history traits and dengue virus loads post-infection. We predicted that larval crowding and high temperatures would both lead to smaller mosquitoes that might struggle to invest in an immune response and, hence, would exhibit high viral loads.

**Methods:**

We first examined larval densities from urban and rural areas via a meta-analysis. We then used these data to inform a laboratory-based 2x2 design examining the interacting effects of temperature (21 vs. 26°C) and density (0.2 vs. 0.4 larvae/mL) on adult life history and dengue virus loads.

**Results:**

We found that urban areas had an ~8-fold increase in larval densities compared to more rural sites. In the lab, we found that crowding had more impact on mosquito traits than temperature. Crowding led to slower development, smaller mosquitoes, less survival, lower fecundity, and higher viral loads, as predicted. The higher temperature led to faster development, reduced fecundity, and lower viral loads. The virus-reducing effect of higher temperature rearing was, however, overwhelmed by the impact of larval crowding when both factors were present.

**Conclusions:**

These data reveal complex interactions between the environmental effects experienced by immature mosquitoes and adult traits. They especially highlight the importance of crowding with respect to adult viral loads. Together, these data suggest that urban environments might enhance dengue virus loads and, therefore, possibly transmission, a concerning result given the increasing rates of urbanization globally.

## Author summary

The mosquito *Aedes aegypti* is responsible for transmitting several viruses to humans, including Zika, yellow fever, chikungunya, and dengue. The geographic distribution of this tropical, anthropophilic mosquito vector continues to expand globally due to climate change and increasing urbanization. Ambient temperature and larval densities are two of the most powerful factors in the aquatic juvenile stage in determining adult mosquito traits. Using a meta-analysis, we reveal that urban environments are associated with greater crowding in the larval phase for *Ae*. *aegypti*. In the laboratory, we then demonstrate that similar levels of larval crowding also led to increased dengue virus loads in adults following an infectious bloodmeal, which may increase transmission. In contrast, higher temperatures in the larval phase were associated with reduced viral loads in adults, but only when crowding is not present. This is a concerning relationship given that 68% of the world’s population will live in urban environments by 2050.

## Introduction

Mosquito-borne diseases like dengue fever [1], chikungunya [2,3], and Zika [4] are rising globally due in part to the expanded range of their vectors, *Aedes aegypti* and *Aedes albopictus*. By 2050, half of the world’s population will likely live in association with one of these species [5]. *Ae*. *aegypti*, is especially anthropophilic, thriving in urban areas, where it breeds in artificial containers (buckets, plant trays, air conditioning units, etc.) inside and near human dwellings (reviewed in [6]). The combination of increased urbanization, human-meditated dispersal, and climate change is expanding mosquito ranges [5]. The implications of these expansions for virus transmission and human disease burdens are not fully understood [5,7,8] but will necessitate shifts in local needs for vector control and pathogen-specific vaccine and diagnostic deployment.

For organisms with complex life cycles, like mosquitoes, the conditions experienced in the larval phase can carry-over to affect the condition and performance (i.e. the phenotype) of the adult. These phenotypic links between life history stages can be as important as demographic links, shaping the dynamics of the adult populations and, in the case of vectors, the disease burdens they impose. The two most critical components of the larval environment are temperature and larval density. As ectotherms, temperature is a fundamental modifier of mosquito physiology, with higher temperatures speeding larval development rates and reducing adult size [9]. Similarly, high larval densities reduce nutrient availability in larval habitats (typically artificial containers) that are already frequently nutrient-poor [10], extending development and, again, reducing adult size. Regardless of the stressor, small adults exhibit reduced fecundity and survival with concomitant effects on vector dynamics [11–13].

With respect to the ability of a mosquito to transmit viruses (vector competence), stress experienced in the larval stage matters. On one hand, smaller females, resulting from low larval nutrition, may seek more repeated bloodmeals in the field with a greater cumulative lifetime potential for pathogen transmission [14–16]. On the other hand, larger females may consume more virus during a single blood meal, increasing their probability of infection [17]. For *Ae*. *aegypti*, nutritional stress in either mothers or their offspring makes them more likely to transmit Zika virus after a single feeding event [18], exhibiting higher viral loads in their saliva. [19]. For dengue virus, the consequences of the larval environment for transmission are unclear, with different studies making different conclusions (Reviewed in [20,21]). There are several possible explanations as to why nutrition may affect viral transmission. Smaller mosquitoes may have fewer excess resources to spend on mounting a costly immune response [22] that would otherwise slow its progression in the mosquito body or reduce salivary viral loads. Starvation may also affect other aspects of physiology, including the permeability of known barriers to tissue progression for viruses like those in the midgut and the salivary glands [18].

Humans are altering the habitats of *Aedes* via urbanization and global warming [5], both of which are increasing the temperature *Aedes* experience as larvae. Urban heat island effects [23] will likely exacerbate longer-term temperature increases due to climate change. But urbanization could also alter the larval habitats of *Aedes* in more subtle ways, driving changes in larval densities. Urbanized environments may be more degraded, such that the abundance of larval predators (birds, beetles, and heterospecific mosquito larvae) is decreased and/or their composition altered (reviewed in [24]). If urbanized environments release *Aedes* from larval predation, then we might expect systematic differences in larval densities between urban and non-urban habitats [25,26] but formal meta-analyses of the effects of urbanization are lacking. Such differences in larval densities could alter adult phenotypes and affect their vector competence for viruses–such that urban environments enhance infectivity in ways that have not yet been appreciated. Recently generated predictive maps of *Aedes* mosquito distributions [5,27] and pathogen transmission zones [7,8] have been constructed based on evidence of past field collection of mosquitoes, pathogen transmission zones, laboratory measures of thermal performance for viruses and vectors, and abiotic habitat characteristics. These modeling approaches do not yet account for how stressors, specifically in the larval phase, alter pathogen transmission in adults.

Here, we first explore the evidence for differences in larval densities in urban vs. rural habitats using a meta-analysis of the literature. Any such differences, in combination with cities acting as heat sinks, would have potential consequences for adult vector competence along rural-urban gradients. We then carried out a two x two laboratory-based design to test the effects of temperature and *Ae*. *aegypti* density in the larval phase on mosquito life history traits and subsequent adult suitability for dengue virus replication. What is novel in our design is that we have isolated temperature effects in the larvae from those in the adult which confound virus replication. We predicted that higher temperatures and crowding would both produce smaller adult mosquitoes, albeit possibly through two different mechanisms—faster development and stress, respectively. We predicted that the resulting smaller mosquitoes might exhibit reduced survival and fecundity and would invest relatively less energy in immunity, exhibiting higher viral loads. We expected mosquitoes experiencing both crowding and higher temperatures would be more severely affected than mosquitoes experiencing only one of these conditions.

## Materials and methods

### Systematic meta-analysis on mosquito density

To determine whether urban environments harbor higher densities of *Aedes* larvae than non-urban environments, we conducted a systematic meta-analysis. On March 7^th,^ 2023, we followed the Preferred Reporting Items for Systematic Reviews and Meta-Analyses (PRISMA) guidelines [28], and searched the peer-reviewed database, Web of Science–Core Collection, for studies comparing *Aedes* mosquito larval density in urban and non-urban environments (**S1 Fig**). Using the search term (urban* “aedes” aegypti or albopictus larval density), we found 2,112 references to be screened at the title and abstract level. Of the 2,112 references, 526 were subject to full-text screening, 67 met most of our inclusion criteria but failed to report the volume of the containers, and 21 met all of our criteria (**S1 Table**). Both measures are useful for comparing densities in a relative sense, but only the latter can be used to compare densities in an absolute sense.

To be included in our meta-analysis, studies had to 1) report larval densities (or immature stages, such as eggs or pupae) of 2) *Aedes aegypti* and/or *Ae*. *albopictus* 3) in an urban and non-urban environment 4) on a per-container basis. Non-urban environments included rural, suburban, peri-urban, forest, or industrial environments. Studies that did not classify their sites as urban and non-urban were still included if they reported the human population density of their sites; here, we compared the most populated area to the least populated area. We also included studies that reported mosquito larval density some distance from an urban area; in these studies, we compared the closest and furthest locations.

We excluded studies that did not meet the above inclusion criteria and those that confounded habitat type and urban classification. For example, we would exclude a study that surveyed larvae in tires in the urban environment but tree holes in the non-urban environment. We also excluded studies that compared areas based on socioeconomic status alone and studies that experimentally manipulated mosquito densities.

For each urban-non-urban comparison, we recorded the mean larval density, error associated with the mean, and sample size. Data were extracted directly from the text or tables or from figures using WebPlotDigitizer (version 4.6) [29]. From each study, we recorded the mosquito species (i.e., *Ae*. *aegypti* and/or *Ae*. *albopictus*), how larval density was measured (i.e., larvae per liter, eggs per trap), and the urban-non-urban comparison (i.e., urban-rural, urban-suburban, etc.). We gave each study a unique identifier, as multiple effect sizes came from the same study.

We used the log response ratio to determine whether urban environments have higher densities of *Aedes* larvae. The log response ratio is calculated as:

$$lnR=ln(X_{U}/X_{NU})$$ (1)

where X_U_ is the larval density in the urban environment, and X_NU_ is the larval density in the non-urban environment (n _effect sizes_ = 105, n _studies_ = 21).

We calculated the unbiased, standardized mean difference, Hedges’ g, as per Borenstein et al [30], when studies reported the mean, standard deviation, and sample size (n _effect sizes_ = 202, n _studies_ = 67) but did not include a volume for the larval container, and present this in the supplementary material (**S2 Fig**). Hence, we analyzed the 21-study dataset using log response ratios and the 67-study dataset using Hedges’ g. We did not analyze Hedges’ g for the smaller dataset as there were too few studies that reported the necessary components (n = 11).

Hedges’ g and the log response ratio are interpreted as follows: a value of zero means that there is no difference in mosquito density between urban and non-urban environments. A positive value indicates that larval density is greater in urban environments than non-urban environments, while a negative value indicates the opposite.

### Larval rearing and 2x2 experimental design

**Fig 1:**
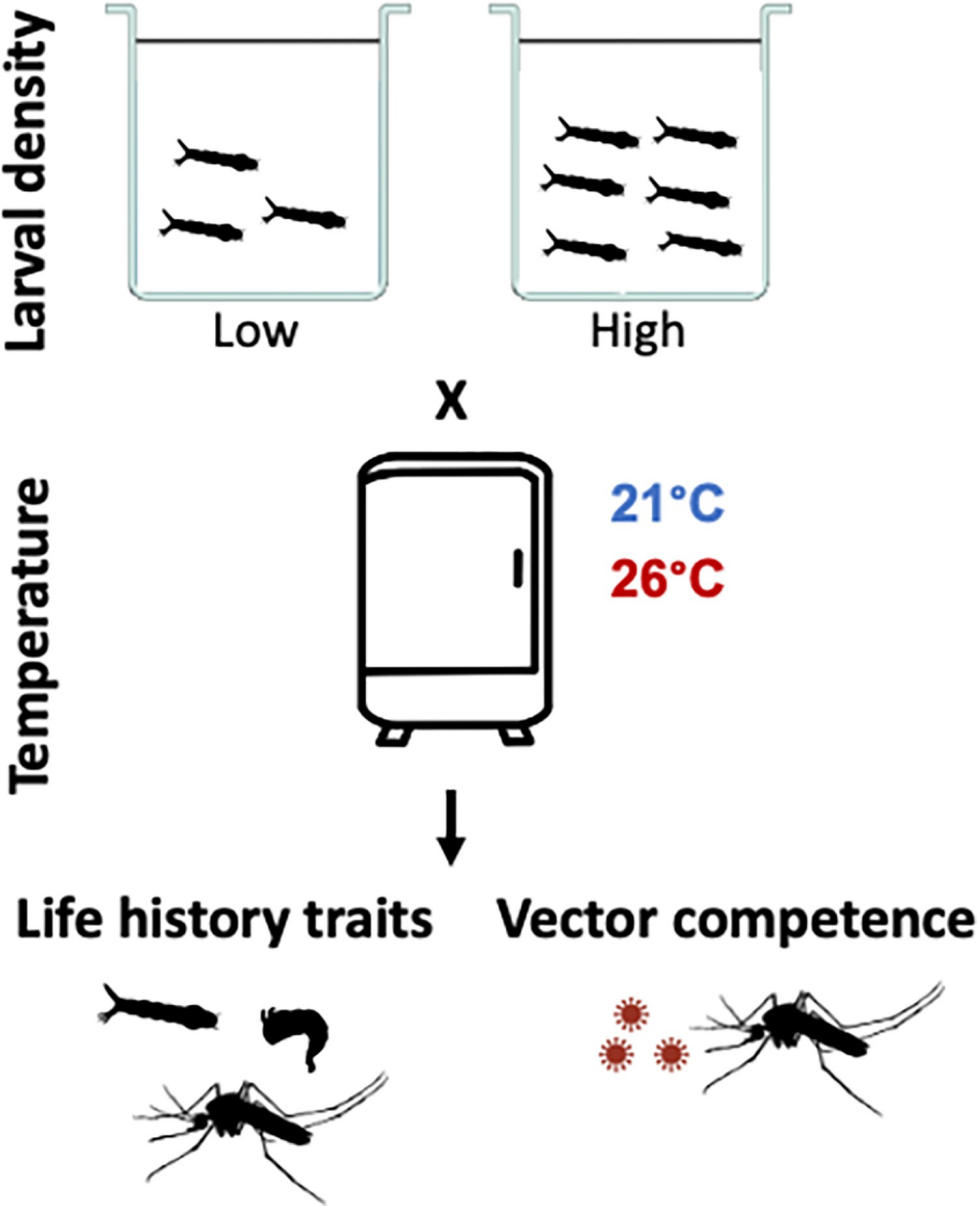
Experimental design for *Aedes aegypti* exposure to temperature and crowding effects (2x2) with replication across incubators in the larval phase followed by measurement of adult life history traits as well as vector competence for dengue virus.

Mosquitoes were derived from a lab-established colony of wildtype *Ae*. *aegypti* mosquitoes previously collected from the field in Mérida, Mexico, by Pablo Manrique-Saide. Eggs were hatched synchronously in separate trays containing filtered, dechlorinated, and oxygen-depleted water for 1h without a food source. After eclosion, first-instar larvae were transferred to 150mL clear hard plastic tumbler cups (6 x 4.5 x 7 cm) containing 100mL of filtered, dechlorinated water, according to their respective density. Larvae were sorted following a 2x2 design (**Fig 1**) consisting of two distinct densities, 20 or 40 larvae per cup, distributed in 20 individual replicate cups across two incubators set at either 21°C or 26°C (12:12h light cycle—70% relative humidity). Twenty larvae/cup is our laboratory’s standard low-density rearing regime that minimizes competition and maximizes adult mosquito size. The number of larvae in the crowded treatment was determined through piloting to find a density that reduced mosquito size but did not extend mean development time >7 days beyond that of low-density treatment to allow comparisons under similar environmental conditions/time windows. The two temperatures were selected to represent the average annual low temperature in Meridá and the optimal temperature for *Ae*. *aegypti* [31]. Rather than test a third treatment level, such as an average of the upper temperatures in Mérida, we opted to expend the experimental effort to test and replicate each condition across a second incubator to reduce any spurious incubator effects that can occur. Regardless of density or temperature treatment, each larval rearing cup was fed approximately 5 mg of ground fish food (Tetramin) daily, which did not vary. This feeding regime (or 0.25 mg food/larva/day) was previously determined as ‘low-stress’ for 20 larvae or the equivalent of our low crowding treatment, as per [32,33]. Our crowded treatment (40 larvae), therefore, received 0.125 mg/larva/day. The previous study employed 0.05 mg/larva/day to represent extreme stress [33], although this lengthened the development time significantly (>20 days).

### Larval development time and survival rate

Larval development, recorded as the day of pupation per individual, was noted daily. Pupae were sexed on emergence by visual analysis of the last segment of their Terminalia using a stereomicroscope. Male and female numbers were recorded, and individuals were placed in cardboard cups (10 x 7.6 x 10cm) covered with mesh. A 10% sucrose solution was offered *ad libitum* and changed daily to avoid mold. Survival rate was calculated as the number of individuals that pupated out of the total number placed in the cup (i.e. 20 larvae/cup).

### Fecundity and weight measurement

Pupae from each of the 20 cups were then transferred to individual cages (10 x 7.6 x 10cm) for emergence and placed at 26°C. All conditions were reared at the same temperature during adulthood. Following mating in the small populations, 5–7 day-old females were offered a bloodmeal of blood from anonymous human donors (BioIVT) using a Hemotek membrane feeding system (Hemotek). Females were deprived of sucrose 12 hours before feeding. Given the differences in developmental rate imposed by the 2x2 regime described above, feeding was first offered to all females from the density of 20 larvae/cup (both temperatures). One week later, females from the 40 larvae/cup density were fed using the same procedure and blood donor. Forty-eight hours post bloodmeal, a total of six females per each one of the twenty cages (120 fecundity measures per treatment) were placed individually in 70mL oviposition cups containing moist filter paper, with access to 10% sucrose to estimate fecundity. Females were allowed to lay eggs for three consecutive days. On the third day, egg papers were collected and manually counted using a stereomicroscope. After collecting egg papers, females were starved for 48-56h, anesthetized, and individually weighed live using an analytical balance.

### Vector competence

To assess the impact of temperature and crowding on mosquito vector competence, we ran a parallel but separate 2x2 design consisting of the same design described above but reducing the number of replicates from twenty to ten. At 5–7 days of adulthood, females were offered an infectious blood meal containing the DENV serotype 2 strain ET300 (GenBank: EF440433). The schedule of blood feeding was the same as above for the fecundity assessment, with the density of 20 larvae/cup feeding a week earlier than females from the 40 larvae/cup treatment. Mosquitoes were offered blood from the same blood donor. Additionally, to avoid using a frozen virus with less activity in both feeding schedules, mosquitoes were offered a freshly harvested normalized viral titer of 3 x 10^7^ viral gene copies/mL per feed (as per below). Fed females were then housed in their respective cages, according to the 2x2 design, until collection. At 14 days post-viral feed, individual females had their legs dissected and stored in 300μL of Trizol (Sigma), then transferred to -80°C until further processing. This collection was done to assess virus transmission potential, as recently shown to be a more accurate method than saliva collection through the capillary method [34].

### Virus culture and quantification

The virus was cultured in *Ae*. *albopictus* C6/36 cells at 26°C in RPMI 1640 medium (Invitrogen, Carlsbad, CA) supplemented with 10% fetal bovine serum (FBS) and HEPES buffer (20mM final concentration). Cells were first allowed to form monolayers of around 60–80% confluence in T-175 flasks (Sigma Aldrich, St. Louis, MO) and then were inoculated with DENV and maintained in RPMI medium supplemented with 2% FBS. At day 7 post-inoculation, live virus was harvested, titrated via absolute quantification RT-qPCR, and adjusted to a final viral load of 10^7^ DENV copies per mL.

All material for virus quantification (supernatant, tissues) was placed in 300 μL of Trizol (Invitrogen) in 1.5 mL microfuge tubes (Sarstedt, Nümbrecht, Germany) and extracted as per the manufacturer’s instructions. A 2.8 mm ceramic bead was added to tissue dissections first, and samples were homogenized on a Bead Ruptor Elite (Omni International, USA) and then frozen at −80°C. RNA was resuspended in 50 μL nuclease-free water and quantified using the NanoDrop 2000 spectrophotometer system (ThermoFisher Scientific). RNA was then treated with 5 units of DNase I (Sigma-Aldrich) at room temperature for 15 min, followed by inactivation with 50 mM EDTA at 70°C for 10 min and stored at -80°C.

DENV virus was quantified using TaqMan Fast Virus 1-step Master Mix (Thermo Fisher Scientific) in 10-μL reaction volumes with DENV-specific primers and probes as per previous [35]. The following protocol was used: reverse transcription at 50°C for 5 min, reverse transcription inactivation at 95°C for 20 sec, followed by 50 amplification cycles of denaturation at 95°C for 30 sec and priming/extension at 60°C for 30 sec. A standard reference curve of known quantities of a DENV-2 genomic fragment was used for absolute quantification by qPCR. The DENV-2 genomic fragment was previously [35] inserted into a plasmid and transformed into *Escherichia coli* as described. The linearized and purified fragment was serially diluted, ranging from 2.5x10^7^ to 2.5x10^3^ copies μL, and used to create a standard curve of DENV amplification. The standard curve was run in duplicate on each 96-well plate, and the limit of detection was set at 10^2^ copies. All samples were run in triplicate.

### Statistical analysis

**Fig 2:**
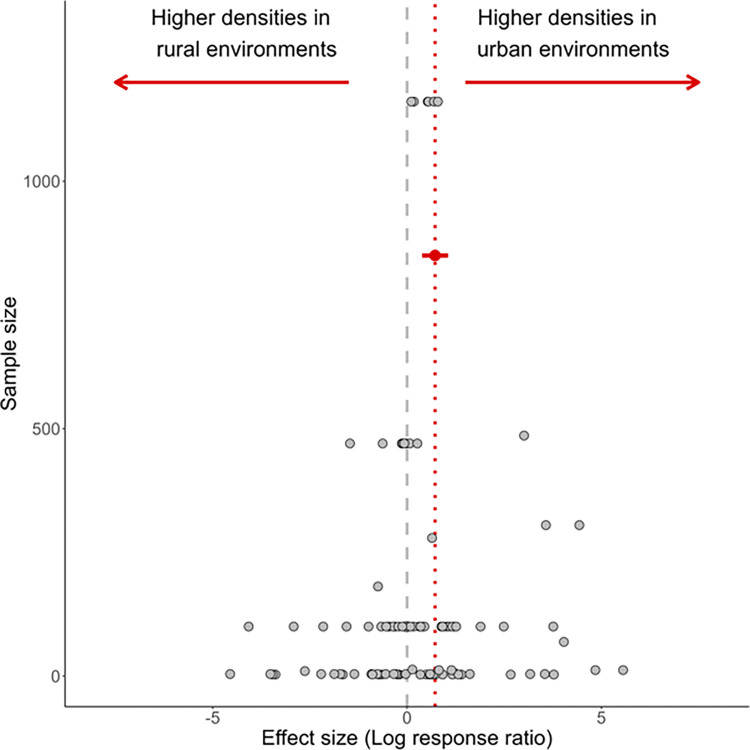
Summary of effect sizes of meta-analysis of the effects of urbanization on larval density in disease vectors. Points to the right of zero indicate that urbanization increased density, points to the left indicate that urbanization decreased larval density. The red dotted line indicates the mean effect size, and the dot with whiskers indicates the mean with 95% confidence intervals. The Y axis indicates the sample size of each study on a log10 scale.

For the meta-analysis, we used a mixed-effects model to determine whether larval density was greater in urban or non-urban environments. We ran two models, one with the log response ratio as the response and one with Hedges’ g as the response. Our models included an intercept and study as a random effect—as multiple effect sizes came from the same study. Including ‘study’ as a random effect accounted for 41% of the variance in the log response ratio model and 17% of the Hedges’ g model. We checked for publication bias by examining funnel plots of the sample size and effect sizes for the log response ratio and of the precision and model residuals for Hedges’ g. The funnel plots were uniformly distributed, indicating no evidence of publication bias (**Figs 2** and **S2**).

We used a mixed-effect model for all variables where Incubation Temperature and Larval Density were fixed effects, Incubator(Temperature) was a random effect, and Experimental Run was a fixed blocking effect. Note that the design was partly nested where different factors are tested over different denominators depending on the scale at which the treatment is applied. Temperature effect were generally tested over the denominator of Incubator(Temperature), density effects were tested of Cup(Temperature x Density), see Quinn and Keough (2002) for further details [36].

## Results

Based on 21 published studies (**S1 Table**), *Aedes* larval density per container in breeding habitats was higher in urban environments than in non-urban environments (mean Log Response Ratio: 0.832, t_20_ = 2.218, p = 0.038, **Fig 2**). On average, larval densities were 8.6 times (±3 SE) higher in urban environments than non-urban environments. For a broader range of studies, where comparisons were made between urban and rural environments, but container size was not specified, we found similar effects based on Hedges’ g (mean Hedges’ g: 0.228, t_66_ = 2.07, P = 0.042). Excluding the most extreme values, the mean densities across the 21 studies were 0.5 larva/mL and 1 larva/mL, for rural and urban, respectively. We saw crowding effects on viral load in our design (above) under less extreme conditions: 0.2 larvae (low density) and 0.4 larvae (high density). In most of the published studies, collections were from tropical or subtropical regions, and while temperature during collections (often over many weeks) was not always reported, the local annual average low to average highs for each ranged from 21–36°C.

To then test the relative importance of larval densities and temperature on adult mosquito life history traits and virus transmissibility by the mosquito, we reared larvae at either crowded (40 larvae per cup) or uncrowded (20 larvae per cup) conditions at two temperatures (21°C or 26°C) (**Fig 1**). Given equal amounts of food, crowding equates to reduced access to nutrients for the higher-density group. We then measured a range of adult traits, including pupation time, sex ratio, weight, fecundity, survival, and vector competence for dengue virus.

**Fig 3:**
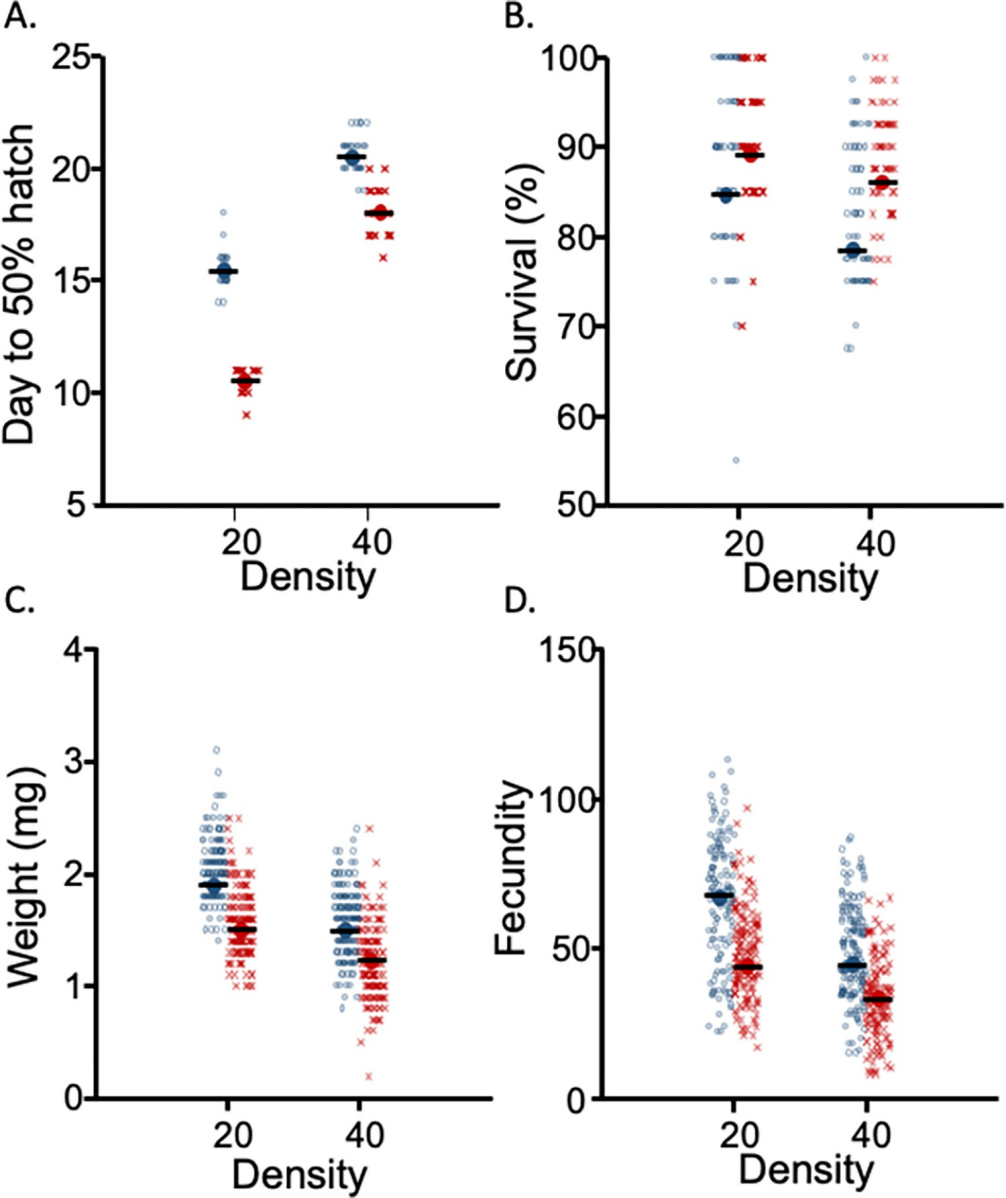
Life history traits for two larval rearing temperatures (blue = 21°C, red = 26°C) and two larval densities (20 vs. 40 individuals). (A) Egg to pupal stage development time in days reported as time to 50% hatch n = 20 per treatment. (B) Survival percentage to the pupal stage. n = 60 per treatment. (C) Adult female total body weight (mg) post 48 hours starvation. N = 20 per treatment. (D) Adult female fecundity, as measured by total eggs produced over three days post blood feeding, is n = 20 per treatment. In all cases, individuals are shown in the figure, but the analysis reflected the number of independent applications of each treatment at the level of cups. The mean is denoted with a bold data marker with a line through it.

For *development time* (time to pupation), both the main effects of temperature (F_1,2_ = 89.20, *p* = 0.011) and larval density (F_1,86_ = 1,089.56, *p* < 0.0001) were significant (**Fig 3A**), as well as their interaction (F_1, 86_ = 26.80, *p* < 0.0001). As predicted, development was slower at lower temperatures. The high-density treatment also slowed development, as hypothesized. Overall, however, density was a greater determinant of hatching time than temperature, averaging a 1.6-fold difference between low and high. For *sex ratio*, populations from lower densities had slightly more (4.5%) males (F_1,76_ = 4.97, *p* = 0.029) than populations reared at higher densities (**S3 Fig**). There was no effect of temperature (F_1, 2_ = 0.82, *p* = 0.46) nor an interaction (F_1,76_ = 0.96, *p* = 0.33). *Survival to adulthood* (**Fig 3B**) was unaffected by larval rearing temperature (F_1,5_ = 3.43, *p* = 0.12), but higher-density rearing led to a decrease (F_1,228_ = 36.07, *p*<0.001) in survival of approximately 5%. Together with the sex ratio data, this may suggest reduced survival in females in the crowded condition. There was no interaction between temperature and density (F_1,228_ = 0.575, P = 0.45). For *adult weight*, density was significant (F_1,76_ = 197.72, *p*<0.001), whereas temperature (F_1,2_ = 12.87, p = 0.07) and the interaction (F_1,76_ = 0.25, *p* = 0.62) were not (**Fig 3C**), although the temperature effect was marginally not significant. Higher density caused a reduction in adult weight by a factor of ~1.3 fold. For *fecundity* (# eggs produced), larval density (F_1,76_ = 130.41, *p*<0.001) and rearing temperature (F_1,2_ = 35.54, *p =* 0.028) were significant (**Fig 3D**), but not their interaction (F_1,76_ = 0.80, *p* = 0.37). The effect of density and temperature was approximately the same: cooler temperatures increased per capita fecundity by 1.4-fold, as did lower densities.

**Fig 4:**
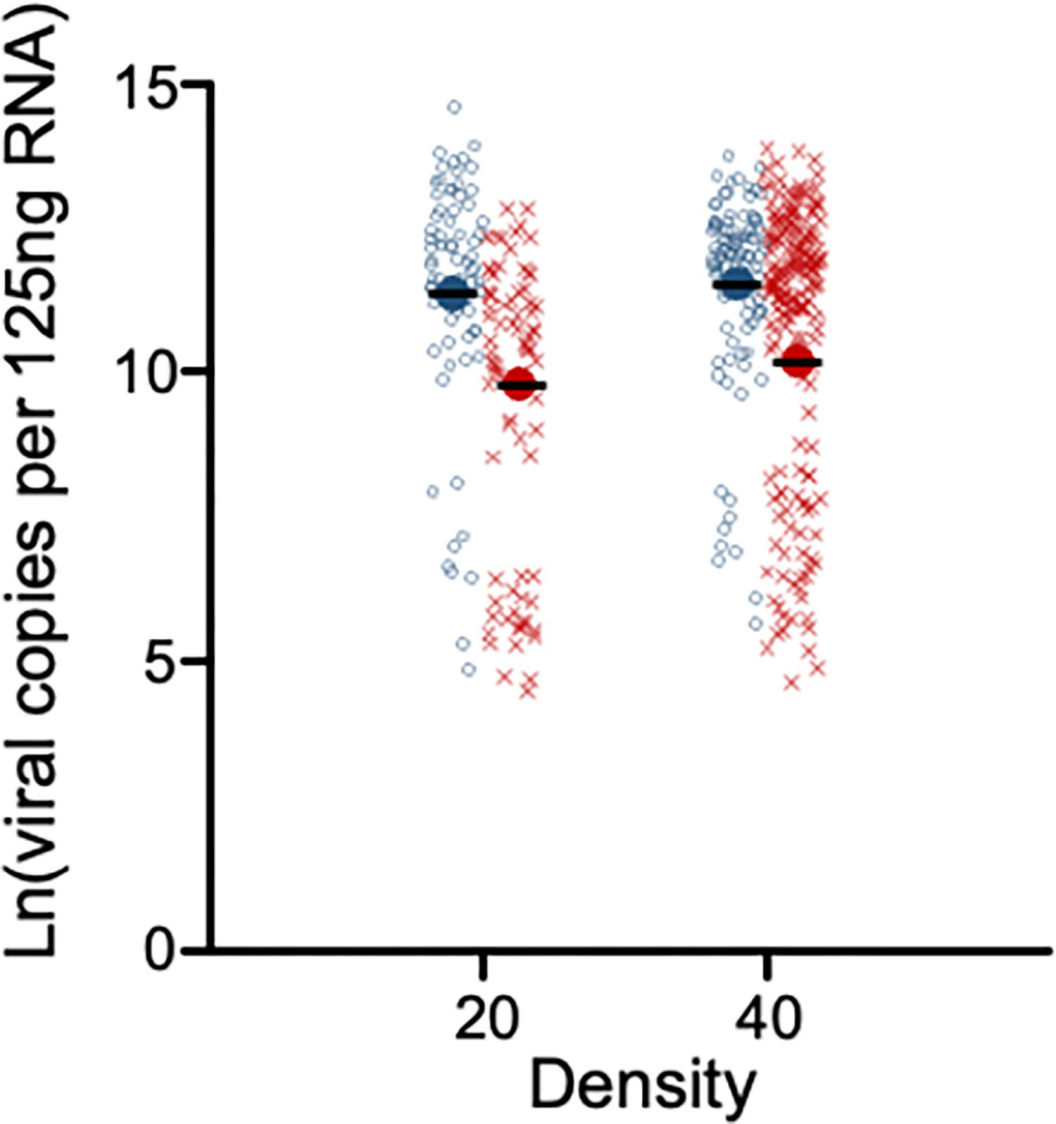
Adult female disseminated viral loads. Dengue virus copy number in leg tissues as determined by qRT-PCR relative to a standard curve and reported per 125 ng RNA for two larval rearing temperatures (blue = 21°C, red = 26°C) and two larval densities (20 vs. 40 individuals). n = 10 per treatment. Individuals are shown in the figure, but the analysis reflected the number of independent applications of each treatment at the level of cups. The mean is denoted with a bold data marker with a line through it.

All adult female mosquitoes were infected with DENV (100% prevalence in all treatments) when feeding on infectious blood. Total body viral loads, our measure of vector competence, however, varied (**Fig 4**) and exhibited a somewhat bimodal distribution, which is common to such studies [37]. Larval density (F_1, 36_ = 7.94, *p* = 0.008) and the interaction with temperature (F_1, 36_ = 5.91, *p* = 0.020) were significant. The main effect of temperature was not (F_1,2_ = 15.73, *p* = 0.058). High-temperature rearing decreased total viral loads in adults on average 4-fold in mosquitoes reared at lower densities and by only 1.2-fold for mosquitoes reared at high densities. In short, the lower temperature and crowding condition increased virus replication, whereas the higher temperature with reduced crowding had the opposite effect. The findings were not as predicted, with the high temperature and crowding conditions acting in opposition, indicating that virus loads cannot be driven purely by body size.

As expected, crowding negatively affected most traits (slower development, reduced female survival, smaller mosquitoes, reduced fecundity, higher viral loads). Mosquitoes reared at the higher temperature did develop more quickly but exhibited fewer other effects except for fecundity, making crowding a more impactful trait determinant. This also held true for viral loads, where even when the higher temperature led to reduced loads, the effect was largely erased by crowded rearing.

## Discussion

Our meta-analysis indicated that urban environments tend to increase larval densities in breeding habitats (by at least 8-fold). Our laboratory work then demonstrated that larval density effects affected all the adult traits we studied and, in some cases, modified the effects of temperature. Larval crowding reduced female survival at the pupal stage, slowed development, and produced smaller adults with reduced fecundity and longevity. Most importantly, we found that larval crowding increased viral loads. The higher temperature had weaker effects than anticipated, speeding development and reducing fecundity but did not produce smaller mosquitoes, although there was a trend in that direction. The higher temperature also resulted in lower disseminated viral loads, counter to expectation, but this only remained true in the absence of larval crowding.

We suspect nutrient limitation under crowding drives the effects of larval crowding on vector competence. Individuals who experience resource limitation in the larval phase may imbibe less blood per sitting [17] or feed more frequently [16] as adults and may also have thinner midgut basal lamina [18]. This tissue barrier limits the dissemination of viruses into the body, and thinning via resource limitation is associated with greater dissemination and higher viral loads in the saliva, which may translate to greater transmission. Larvae with restricted intakes also exhibit changes to their bacterial microbiomes in the adult stage, including lower total abundance and reduced diversity [12]. The microbiome can affect vector competence for viruses [38] via innate immune system activation, direct antiviral action, or resource competition [39]. Larval crowding alters myriad other factors beyond resource intake, many of which could contribute to viral loads. For example, crowding has been shown to affect gene expression in the fat body–a metabolic and fat-storage organ. Of the many pathways whose expression is affected, those involving immunity and autophagy may directly affect viral replication [40].

There are two potential larval pathways to reduced adult size—either by faster development in higher temperatures [41] or slower development in crowded conditions, as seen here. The warmer temperature/faster development yielding smaller adult sizes conforms to the ‘hotter is smaller’ rule common to most animals (reviewed in [42]). In contrast, larvae took longer to develop under crowded conditions but still emerged smaller as adults–an effect that is also well-established in animals [43,44]. Of the two factors we manipulated, crowding had a greater effect on the range of life history traits than temperature and was a stronger determinant of developmental rate. While the opposite has been demonstrated elsewhere [41], the level of crowding may be the greater stressor in our study, with our 26°C degree treatment being a temperature routinely experienced by *Ae*. *aegypti* in its tropical range [5,27] and in the laboratory. We expect exposure to a slightly higher temperature or simply greater replication to enhance power across our study design, which might have yielded a significant relationship between high temperature and reduced size, given the estimates seen here (*p* = 0.071).

For most animal species, urbanization poses challenges. As cities expand and infrastructure develops, natural habitats are fragmented and destroyed, resulting in the loss of critical habitats [45]. Some species adapt to urban environments, thriving in human-altered landscapes and taking advantage of novel food sources and habitats [45,46], while others struggle to adapt, facing population declines and even extinction [47]. *Ae*. *aegypti* is an example of the former, a highly adapted ‘human habitat specialist’ breeding in artificial containers [6] and feeding almost exclusively on humans [48]. The higher larval densities in cities demonstrated by our analysis may be driven by reductions in predators/predator diversity [24], or more frequent access to nutrient-contaminated plastic trash as breeding sites [49], but could also result from the greater reproductive success of females with higher accessibility to hosts [50]. Regardless of the mechanisms, the difference in larval densities between rural and urban densities is remarkably high and may drive increases in viral transmission replication and or transmission potential.

There are several limitations to our study. First, temperature typically has curvilinear effects on life history traits and vector competence traits, exhibiting an optimum, with a poorer performance at higher and lower temperatures [51]. We used only two temperatures because we were interested in exploring the potential synergies between temperature and larval density in a well-replicated design across multiple incubators. Future studies could explore additional temperatures, especially in the warmer ranges, given *Ae*. *aegypti’s* thermal preferences and geographic distribution. In contrast, reduced low resource supply in the larval phase will likely negatively affect vector fitness across all temperatures [52]. Second, the viral load we used in the blood meal produced 100% prevalence in all mosquitoes by the 14-day post-infection assay point, but surveying earlier time points or feeding a lower initial dose may have revealed differences in the treatments. The range of DENV viral load in human blood varies substantially and can also be several orders of magnitude lower than what was used here [53]. We do know from a previous kinetics study where we fed *Ae*. *aegypti* with the same virus strain at two different titers (low -10^5^/mL vs. high- 10^8^/mL), that the former led to lower viral loads in the midgut and carcass and resulted in lower infection prevalence (70 vs 100% at day 14, respectively) in the salivary glands [37]. This suggests that substantial differences in viral load in intermediary tissues also correlate with prevalence or infectivity in the salivary glands, ie vector competence. Additionally, repeated sampling across earlier time points could shed light on both the extrinsic incubation period and early infection prevalence estimates that will be less than zero. Having these two additional parameters, as well as measures of adult survival, would allow for the estimation of vectorial capacity under the Ross-MacDonald equation [54], encompassing some of the intrinsic effects [55] of growth under our temperature x density conditions. Similarly, the additional inclusion of adult survival and egg clutch timing would allow for more holistic interpretations of population growth, or *r* [56].

## Conclusions

With increasing global temperatures and urbanization expected in the future [5,27], temperature and larval density effects will increasingly interact over *Ae*. *aegypti’s* geographic range. Urban heat sink phenomena will only further exacerbate temperature effects in cities [23]. Our findings indicate, however, that while higher temperatures in the larval phase might mitigate viral loads in adults, larval crowding can overwhelm these effects, increasing viral loads and possibly transmissibility. This study illustrates that focusing on single global change stressors in isolation may yield inaccurate predictions of the transmission potential of vectors.

## Supporting information

S1 Fig PRISMA flow diagram showing the search and screening process. (TIFF)

S2 Fig The effect of urbanization on *Aedes* larval density (Hedges’ g). The dotted-red line is the mean effect size, which is also represented by the solid-red dot with 95% confidence intervals. The light grey points represent the individual effect sizes. The dashed grey line represents an effect size of zero where there is no difference in larval densities between urban and non-urban environments. Points to the left of this line indicate higher larval densities in non-urban environments than in urban environments. Points to the right indicate higher larval densities in urban environments than in non-urban environments. Although the mean sits to the right of the dashed line, indicating a trend towards more mosquitoes in urban environments, the confidence interval overlaps zero, indicating no difference in larval densities between urban and non-urban environments (χ^2^_1_ = 1.294, p = 0.255, n _effect sizes_ = 79, n _studies_ = 11). Given the small sample size, we default to the log response ratio data presented in the main text.
(TIFF)

S3 Fig % male recorded at the pupal stage for two larval rearing temperatures (blue = 21°C, red = 26°C) and two larval densities (20 vs. 40 individuals). n = 60 per treatment. Individuals are shown in the figure, but the analysis reflected the number of independent applications of each treatment at the level of cups. Mean designated with horizontal black line.
(TIFF)

S1 Table A summary of the studies used in the meta-analysis. (XLSX)

## References

[1] Bhatt S, Gething PW, Brady OJ, Messina JP, Farlow AW, Moyes CL, et al. The global distribution and burden of dengue. Nature. 2013;496: 504–507. doi: 10.1038/nature12060 23563266 PMC3651993

[2] Leparc-Goffart I, Nougairede A, Cassadou S, Prat C, De Lamballerie X. Chikungunya in the Americas. The Lancet. 2014;383: 514. doi: 10.1016/S0140-6736(14)60185-9 24506907

[3] Weaver SC, Forrester NL. Chikungunya: Evolutionary history and recent epidemic spread. Antiviral Res. 2015;120: 32–39. doi: 10.1016/j.antiviral.2015.04.016 25979669

[4] Weaver SC, Charlier C, Vasilakis N, Lecuit M. Zika, chikungunya, and other emerging vector-borne viral diseases. Annu Rev Med. 2018;69: 395–408. doi: 10.1146/annurev-med-050715-105122 28846489 PMC6343128

[5] Kraemer MUG, Reiner Jr. RC, Brady OJ, Messina JP, Gilbert M, Pigott DM, et al. Past and future spread of the arbovirus vectors *Aedes aegypti* and *Aedes albopictus*. Nat Microbiol. 2019;4: 854–863. 30833735 10.1038/s41564-019-0376-y PMC6522366

[6] Flaibani N, Pérez AA, Barbero IM, Burroni NE. Different approaches to characterize artificial breeding sites of *Aedes aegypti* using generalized linear mixed models. Infect Dis Poverty. 2020;9: 1–11. 32736584 10.1186/s40249-020-00705-3 PMC7393697

[7] Ryan SJ, Carlson CJ, Mordecai EA, Johnson LR. Global expansion and redistribution of Aedes-borne virus transmission risk with climate change. PLOS Negl Trop Dis. 2019;13: e0007213. doi: 10.1371/journal.pntd.0007213 30921321 PMC6438455

[8] Mordecai EA, Ryan SJ, Caldwell JM, Shah MM, LaBeaud AD. Climate change could shift disease burden from malaria to arboviruses in Africa. Lancet Planet Health. 2020;4: e416–e423. doi: 10.1016/S2542-5196(20)30178-9 32918887 PMC7490804

[9] Rueda LM, Patel KJ, Axtell RC, Stinner RE. Temperature-dependent development and survival rates of *Culex quinquefasciatus* and *Aedes aegypti* (Diptera: Culicidae). J Med Entomol. 1990;27: 892–898. 2231624 10.1093/jmedent/27.5.892

[10] Washburn JO. Regulatory factors affecting larval mosquito populations in container and pool habitats: implications for biological control. J Am Mosq Control Assoc. 1995; 279–283. 7595462

[11] Briegel H. Metabolic relationship between female body size, reserves, and fecundity of *Aedes aegypti*. J Insect Physiol. 1990;36: 165–172.

[12] MacLeod HJ, Dimopoulos G, Short SM. Larval diet abundance influences size and composition of the midgut microbiota of *Aedes aegypti* mosquitoes. Front Microbiol. 2021;12: 645362. 34220739 10.3389/fmicb.2021.645362 PMC8249813

[13] Nørgaard LS, Álvarez-Noriega M, McGraw E, White CR, Marshall DJ. Predicting the response of disease vectors to global change: The importance of allometric scaling. Glob Chang Biol. 2022;28: 390–402. doi: 10.1111/gcb.15950 34674354

[14] Armstrong PM, Ehrlich HY, Magalhaes T, Miller MR, Conway PJ, Bransfield A, et al. Successive bloodmeals enhance virus dissemination within mosquitoes and increase transmission potential. Nat Microbiol. 2020;5: 239–247. doi: 10.1038/s41564-019-0619-y 31819213 PMC7199921

[15] Brackney DE, LaReau JC, Smith RC. Frequency matters: How successive feeding episodes by blood-feeding insect vectors influences disease transmission. PLOS Pathog. 2021;17: e1009590. doi: 10.1371/journal.ppat.1009590 34111228 PMC8191993

[16] Klowden MJ, Lea AO. Blood meal size as a factor affecting continued host-seeking by *Aedes aegypti* (L.). Am J Trop Med Hyg. 1978;27: 827–831. 686250 10.4269/ajtmh.1978.27.827

[17] Kang DS, Alcalay Y, Lovin DD, Cunningham JM, Eng MW, Chadee DD, et al. Larval stress alters dengue virus susceptibility in *Aedes aegypti* (L.) adult females. Acta Trop. 2017;174: 97–101. 28648790 10.1016/j.actatropica.2017.06.018 PMC5571755

[18] Herd CS, Grant DG, Lin J, Franz AWE. Starvation at the larval stage increases the vector competence of *Aedes aegypti* females for Zika virus. PLOS Negl Trop Dis. 2021;15: e0010003. 34843483 10.1371/journal.pntd.0010003 PMC8659361

[19] Zirbel K, Eastmond B, Alto BW. Parental and offspring larval diets interact to influence life-history traits and infection with dengue virus in *Aedes aegypti*. R Soc Open Sci. 2018;5: 180539. 30109101 10.1098/rsos.180539 PMC6083674

[20] Carvajal-Lago L, Ruiz-López MJ, Figuerola J, Martínez-de la Puente J. Implications of diet on mosquito life history traits and pathogen transmission. Environ Res. 2021;195: 110893. doi: 10.1016/j.envres.2021.110893 33607093

[21] Weger-Lucarelli J, Auerswald H, Vignuzzi M, Dussart P, Karlsson EA. Taking a bite out of nutrition and arbovirus infection. PLOS Negl Trop Dis. 2018;29: e0006247. doi: 10.1371/journal.pntd.0006247 29596427 PMC5875747

[22] Sigle LT, McGraw EA. Expanding the canon: Non-classical mosquito genes at the interface of arboviral infection. Insect Biochem Mol Biol. 2019;109: 72–80. doi: 10.1016/j.ibmb.2019.04.004 30970277

[23] Zhao L, Lee X, Smith RB, Oleson K. Strong contributions of local background climate to urban heat islands. Nature. 2014;511: 216–219. doi: 10.1038/nature13462 25008529

[24] Bonds JAS, Collins CM, Gouagna LC. Could species-focused suppression of *Aedes aegypti*, the yellow fever mosquito, and *Aedes albopictus*, the tiger mosquito, affect interacting predators? An evidence synthesis from the literature. Pest Manag Sci. 2022;78: 2729–2745. 35294802 10.1002/ps.6870 PMC9323472

[25] Isaksson C. Impact of urbanization on birds. 2018; 235–257.

[26] Magura T, Mizser S, Horváth R, Nagy DD, Tóth M, Csicsek R, et al. Differences in life history traits in rural vs. Urban populations of a specialist ground beetle, *Carabus convexus*. Insects. 2021;12: 540. 34200777 10.3390/insects12060540 PMC8230416

[27] Kraemer MU, Sinka ME, Duda KA, Mylne AQ, Shearer FM, Barker CM, et al. The global distribution of the arbovirus vectors *Aedes aegypti* and *Ae*. *albopictus*. Elife. 2015/07/01. 2015;4: e08347. 26126267 10.7554/eLife.08347 PMC4493616

[28] Page MJ, McKenzie JE, Bossuyt PM, Boutron I, Hoffmann TC, Mulrow CD, et al. The PRISMA 2020 statement: an updated guideline for reporting systematic reviews. BMJ. 2021;372. 10.1136/bmj.n71 PMC8005924 33782057

[29] Rohatgi A. WebPlotDigitizer. Pacifica, California; 2022.

[30] Borenstein M, Hedges L V., Higgins JPT, Rothstein HR. Introduction to Meta-Analysis. John Wiley & Sons, Ltd; 2011.

[31] Brady OJ, Johansson MA, Guerra CA, Bhatt S, Golding N, Pigott DM, et al. Modelling adult *Aedes aegypti* and *Aedes albopictus* survival at different temperatures in laboratory and field settings. Parasit Vectors. 2013;6: 351. 24330720 10.1186/1756-3305-6-351 PMC3867219

[32] Dutra HLC, Da Silva VL, Da Rocha Fernandes M, Logullo C, Maciel-De-Freitas R, Moreira LA. The influence of larval competition on Brazilian Wolbachia-infected *Aedes aegypt*i mosquitoes. Parasit Vectors. 2016;9: 282. 27183820 10.1186/s13071-016-1559-5 PMC4869337

[33] Yeap HL, Mee P, Walker T, Weeks AR, O’Neill SL, Johnson P, et al. Dynamics of the “‘Popcorn’” *Wolbachia* infection in outbred *Aedes aegypti* informs prospects for mosquito vector control. Genetics. 2011;187: 583–595. 21135075 10.1534/genetics.110.122390 PMC3030498

[34] Gloria-Soria A, Brackney DE, Armstrong PM. Saliva collection via capillary method may underestimate arboviral transmission by mosquitoes. Parasit Vectors. 2022;15. 35331315 10.1186/s13071-022-05198-7 PMC8944160

[35] Ford SA, Allen SL, Ohm JR, Sigle LT, Sebastian A, Albert I, et al. Selection on Aedes aegypti alters *Wolbachia*-mediated dengue virus blocking and fitness. Nat Microbiol. 2019;4: 1832–1839. 31451771 10.1038/s41564-019-0533-3 PMC6990461

[36] Quinn GP, Keough MJ. Experimental design and data analysis for biologists. Cambridge University Press; 2002.

[37] Novelo M, Hall MD, Pak D, Young PR, Holmes EC, McGraw EA. Intra-host growth kinetics of dengue virus in the mosquito *Aedes aegypti*. PLOS Pathog. 2019;15: e1008218. 31790509 10.1371/journal.ppat.1008218 PMC6907869

[38] Ramirez JL, Souza-Neto J, Cosme RT, Rovira J, Ortiz A, Pascale JM, et al. *Reciprocal* tripartite interactions between the *Aedes aegypti* midgut microbiota, innate immune system and dengue virus influences vector competence. PLOS Negl Trop Dis. 2012;6: e1561. 22413032 10.1371/journal.pntd.0001561 PMC3295821

[39] Ferreira QR, Lemos FFB, Moura MN, Nascimento JO de S, Novaes AF, Barcelos IS, et al. Role of the microbiome in Aedes spp. vector competence: what do we know? Vir. 2023;15: 779. doi: 10.3390/v15030779 36992487 PMC10051417

[40] Price DP, Schilkey FD, Ulanov A, Hansen IA. Small mosquitoes, large implications: crowding and starvation affects gene expression and nutrient accumulation in *Aedes aegypti*. Parasit Vectors. 2015;8: 252. 25924822 10.1186/s13071-015-0863-9 PMC4415286

[41] Couret J, Benedict MQ. A meta-analysis of the factors influencing development rate variation in *Aedes aegypti* (Diptera: Culicidae). BMC Ecol. 2014;14: 3. 24495345 10.1186/1472-6785-14-3 PMC3916798

[42] Reiskind MH, Zarrabi AA. Is bigger really bigger? Differential responses to temperature in measures of body size of the mosquito, *Aedes albopictus*. J Insect Physiol. 2012;58: 911–917. 22543181 10.1016/j.jinsphys.2012.04.006

[43] Pooraiiouby R, Sharma A, Beard J, Reyes J, Nuss A, Gulia-Nuss M. Nutritional quality during development alters insulin-like peptides’ expression and physiology of the adult yellow fever mosquito, *Aedes aegypti*. Insects. 2018;9: 110. 30200185 10.3390/insects9030110 PMC6163675

[44] Yan J, Kibech R, Stone CM. Differential effects of larval and adult nutrition on female survival, fecundity, and size of the yellow fever mosquito, *Aedes aegypti*. Front Zool. 2021;18: 10. 33750400 10.1186/s12983-021-00395-z PMC7941737

[45] McDonnell MJ, Hahs AK. Adaptation and adaptedness of organisms to urban environments. Annu Rev Ecol Evol Syst. 2015;46: 261–280.

[46] Morelli F, Beim M, Jerzak L, Jones D, Tryjanowski P. Can roads, railways and related structures have positive effects on birds?—A review. Transp Res D Transp Environ. 2014;30: 21–31.

[47] McKinney ML. Urbanization as a major cause of biotic homogenization. Biol Conserv. 2006;127: 247–260.

[48] Ponlawat A, Harrington LC. Blood Feeding patterns of Aedes aegypti and Aedes albopictus in Thailand. J Med Entomol. 2005;42: 844–849. doi: 10.1093/jmedent/42.5.844 16363170

[49] Khan A, Bisanzio D, Mutuku F, Ndenga B, Grossi-Soyster EN, Jembe Z, et al. Spatiotemporal overlapping of dengue, chikungunya, and malaria infections in children in Kenya. BMC Infect Dis. 2023;23: 183. doi: 10.1186/s12879-023-08157-4 36991340 PMC10053720

[50] Sun H, Jit M, Cook AR, Carrasco LR, Dickens BL. Determining environmental and anthropogenic factors which explain the global distribution of *Aedes aegypti* and *Ae*. *albopictus*. BMJ Glob Health. 2018;3: e000801. 10.1136/bmjgh-2018-000801 PMC6135425 30233829

[51] Mordecai EA, Cohen JM, Evans M V, Gudapati P, Johnson LR, Lippi CA, et al. Detecting the impact of temperature on transmission of Zika, dengue, and chikungunya using mechanistic models. PLOS Negl Trop Dis. 2017/04/28. 2017;11: e0005568. doi: 10.1371/journal.pntd.0005568 28448507 PMC5423694

[52] Huxley PJ, Murray KA, Pawar S, Cator LJ. The effect of resource limitation on the temperature dependence of mosquito population fitness. Proc Biol Sci. 2021;288: 20203217. doi: 10.1098/rspb.2020.3217 33906411 PMC8079993

[53] Lam Vuong N, Than N, Quyen H, Thi N, Tien H, Tuan NM, et al. Higher plasma viremia in the febrile phase is associated with adverse dengue outcomes irrespective of infecting serotype or host immune status: an analysis of 5642 Vietnamese cases. Clin Infect Dis. 2021;72: e1074–1083. doi: 10.1093/cid/ciaa1840 33340040 PMC8204785

[54] Moller-Jacobs LL, Murdock CC, Thomas MB. Capacity of mosquitoes to transmit malaria depends on larval environment. Parasit Vectors. 2014;7: 593. doi: 10.1186/s13071-014-0593-4 25496502 PMC4273441

[55] Hardy JL, Houk EJ, Kramer LD, Reeves WC. Intrinsic factors affecting vector competence of mosquitoes for arboviruses. Annu Rev Entomol. 1983;28: 229–62. doi: 10.1146/annurev.en.28.010183.001305 6131642

[56] Neale JT, Juliano SA. Predation yields greater population performance: What are the contributions of density- and trait-mediated effects? Ecol Entomol. 2021;46: 56–65. doi: 10.1111/een.12940 34092899 PMC8171192

