# Supplementary figures and images for: Larval crowding enhances dengue virus loads in Aedes aegypti, a relationship that might increase transmission in urban environments
Source: PLoS Negl Trop Dis. 2024 Sep 10;18(9):e0012482. doi: 10.1371/journal.pntd.0012482 (PMC11414913; doi:10.1371/journal.pntd.0012482)

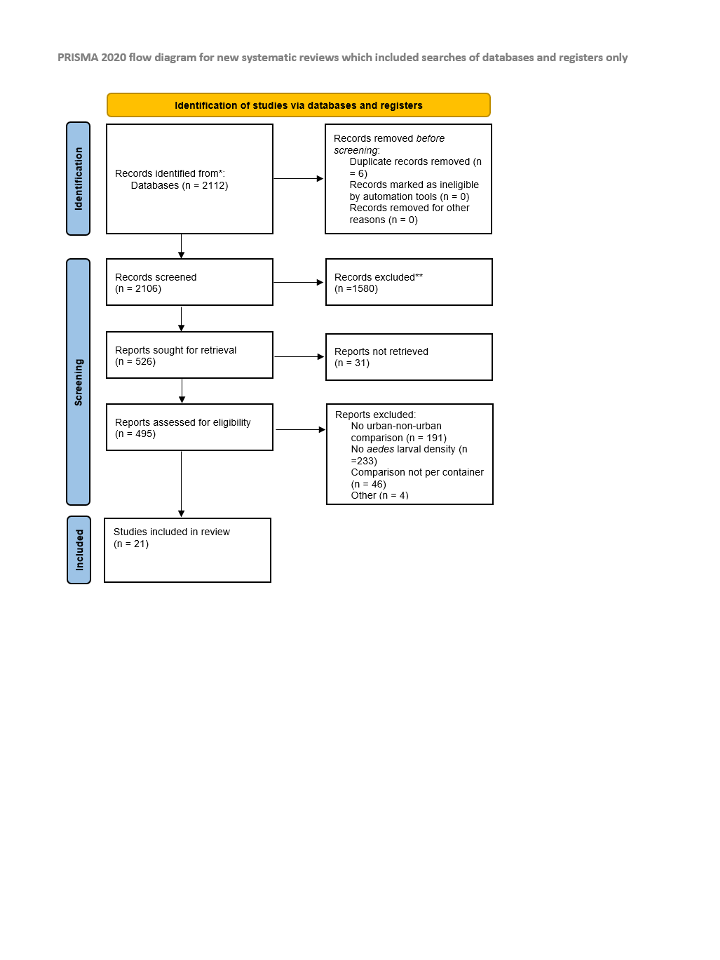

Supplement: S1 Fig — (TIFF) [file pntd.0012482.s001.tiff]

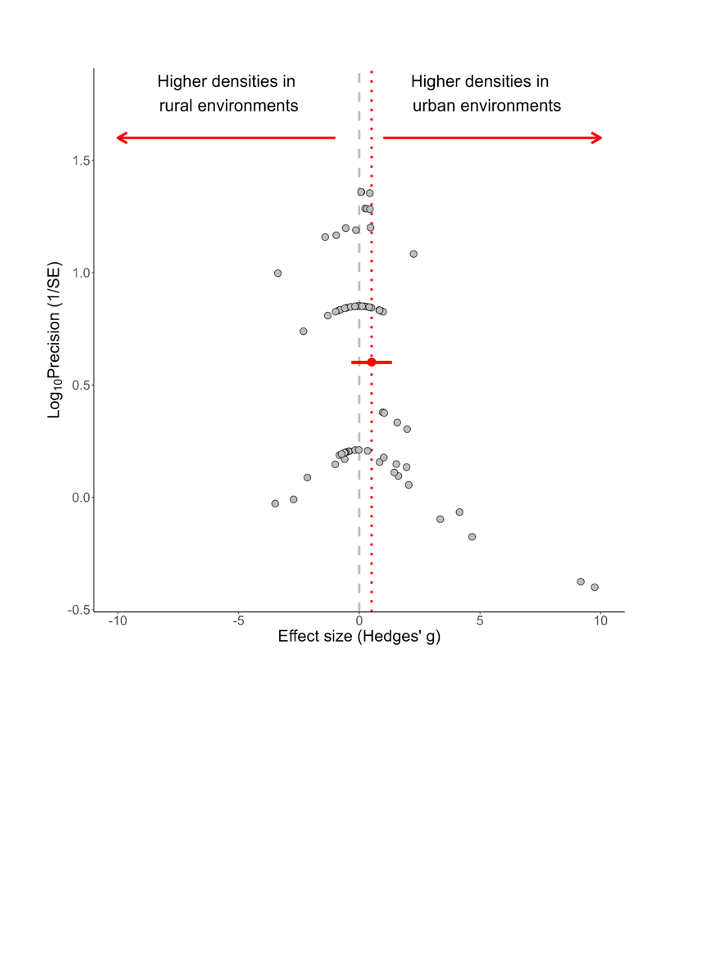

Supplement: S2 Fig — The dotted-red line is the mean effect size, which is also represented by the solid-red dot with 95% confidence intervals. The light grey points represent the individual effect sizes. The dashed grey line represents an effect size of zero where there is no difference in larval densities between urban and non-urban environments. Points to the left of this line indicate higher larval densities in non-urban environments than in urban environments. Points to the right indicate higher larval densities in urban environments than in non-urban environments. Although the mean sits to the right of the dashed line, indicating a trend towards more mosquitoes in urban environments, the confidence interval overlaps zero, indicating no difference in larval densities between urban and non-urban environments (χ21 = 1.294, p = 0.255, n effect sizes = 79, n studies = 11). Given the small sample size, we default to the log response ratio data presented in the main text. (TIFF) [file pntd.0012482.s002.tiff]

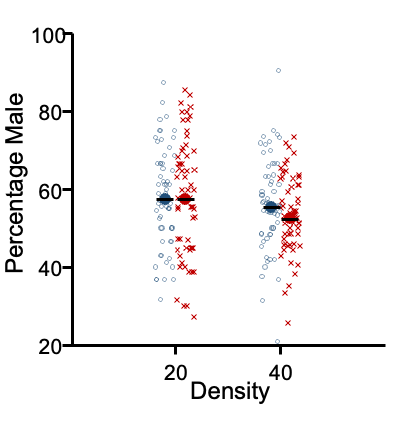

Supplement: S3 Fig — n = 60 per treatment. Individuals are shown in the figure, but the analysis reflected the number of independent applications of each treatment at the level of cups. Mean designated with horizontal black line. (TIFF) [file pntd.0012482.s003.tiff]
